# A230 DEATH, MITOCHONDRIA, AND INFLAMMATION IN *E. COLI*-LF82 INFECTION

**DOI:** 10.1093/jcag/gwae059.230

**Published:** 2025-02-10

**Authors:** A Mohan, A Wang, D McKay, T E Shutt

**Affiliations:** University of Calgary, Calgary, AB, Canada; University of Calgary, Calgary, AB, Canada; University of Calgary, Calgary, AB, Canada; University of Calgary, Calgary, AB, Canada

## Abstract

**Background:**

The Crohn’s disease-associated adherent invasive *E. coli* (AIEC) (strain LF82) decreases mitochondrial membrane potential and fragments the epithelial mitochondrial network. Other groups have also reported activation of NF-κB-mediated inflammation and generation of reactive oxygen species in AIEC-LF82 infected epithelia. It remains unclear how these findings integrate over the course of AIEC-LF82 infection and how they influence each other over the course of infection.

**Aims:**

To characterize the sequence of events following AIEC-LF82 infection in a non-cancer derived epithelial cell model focusing on mitochondrial disruption, cell death, mtDNA release, and inflammatory gene expression.

**Methods:**

The fetal derived human colon epithelial cell line (FHC) were infected with *E. coli*-LF82 or the commensal *E. coli*-HB101 at multiplicity of infection (MOI) of 100 or uninfected over the course of various experiments. FHCs were stained with SYTOX green and imaged on a CellCyte X system for 24h to measure cell death or stained with MitoTracker Deep Red and imaged on a spinning disc-confocal microscope for 16h to assess mitochondrial morphology. Also, in fixed-cell preparations mitochondrial networks were imaged by staining with anti-TOMM20 antibodies. Imaging of cytosolic DNA was performed via immunofluorescent staining of mtDNA and the mitochondrial network. As markers of inflammation, *CXCL10* and *IL-6* mRNA were assessed via qRT-PCR.

**Results:**

Epithelia infected with *E. coli*-LF82 but not *E. coli*-HB101 displayed dramatic fragmentation of their mitochondrial network at 6h (p < 0.0001) (n=6), while SYTOX green imaging revealed significant cell death beginning at 9h (p < 0.001) (n=3). Furthermore, our preliminary data indicates that transcription of inflammatory cytokines including *CXCL10*, and *IL-6* occurs at 8h while cytosolic escape of mtDNA occurs at 6h, after mitochondrial network disruption, but prior to cell death.

**Conclusions:**

The data begins to unravel the sequence of events in a non-cancer derived epithelium after infection with *E. coli*-LF82, integrating mitochondrial function, inflammatory gene expression, and cell viability and providing a more fulsome understanding of the course of *E. coli-LF82* infection. Together, these data advance understanding of relationship between mitochondrial fragmentation and cell death in the context of a Crohn’s disease-relevant pathobiont, AIEC-LF82.

**Funding Agencies:**

TRIANGLE

